# Cellular and molecular mechanisms of highly active mesenchymal stem cells in the treatment of senescence of rhesus monkey ovary

**DOI:** 10.1186/s13287-023-03631-x

**Published:** 2024-01-08

**Authors:** Kai Wang, Xiang Yao, Shu-qian Lin, Xiang-qing Zhu, Xing-hua Pan, Guang-ping Ruan

**Affiliations:** 1 The Basic Medical Laboratory of 920, Hospital of Joint Logistics Support Force of PLA, Kunming, 650032 Yunnan China; 2 The Integrated Engineering Laboratory of Cell Biological Medicine of State and Regions, Kunming, 650032 Yunnan China; 3 The Transfer Medicine Key Laboratory of Cell Therapy Technology of Yunan Province, Kunming, 650032 Yunnan China; 4 Kunming Medical University, Kunming, 650500 Yunnan China

**Keywords:** Highly active mesenchymal stem cells, Ovarian granulosa cells, Ovarian senescence, 10X Genomics single nuclear transcriptome sequencing, Macaque

## Abstract

**Background:**

Recent studies have shown that umbilical cord mesenchymal stem cells have an anti-aging effect in ovaries, but the cellular and molecular mechanisms of HA-MSC ovarian anti-aging remain to be studied. Therefore, we conducted a 10X Genomics single-nucleus transcriptome sequencing experiment on the ovaries of macaque monkeys after HA-MSC treatment.

**Methods:**

The results of cell subgroup classification were visualized by 10X Genomics single nuclear transcriptome sequencing. The aging model of hGCs was established, and the migration ability of the cells was determined after coculture of HA-MSCs and aging hGCs. The genes screened by single nuclear transcriptional sequencing were verified in vitro by qPCR.

**Results:**

Compared with the aging model group, the number of cell receptor pairs in each subgroup of the HA-MSC-treated group increased overall. Treatment with 200 μmol/L H_2_O_2_ for 48 h was used as the optimum condition for the induction of hGC senescence. After coculture of noncontact HA-MSCs with senescent hGCs, it was found that HA-MSCs can reverse the cell structure, proliferation ability, senescence condition, expression level of senescence-related genes, and expression level of key genes regulating the senescence pathway in normal hGCs.

**Conclusions:**

HA-MSC therapy can improve the tissue structure and secretion function of the ovary through multiple cellular and molecular mechanisms to resist ovarian aging. In vitro validation experiments further supported the results of single-cell sequencing, which provides evidence supporting a new option for stem cell treatment of ovarian senescence.

## Background

As the female gonad, the ovary is the core reproductive organ in women. It can produce egg cells and secrete sex hormones, so it plays a very important role in maintaining the normal operation of the reproductive system and stabilizing endocrine function [1]. There are approximately 1 million–2 million follicles in the ovary at birth. With increasing age, only approximately 1000 original follicles remain at the time of menopause. The ovary is one of the first organs in the body to show signs of aging, and compared with other organs, the ovary ages very fast [2]. As a special type of organ aging, the aging of female ovaries not only leads to a decline in endocrine and reproductive functions but also may lead to and aggravate the dysfunction of multiple organs and systems, such as body weight gain, dry skin, loss of skin elasticity, reduced immunity, increased incidence of cervical spondylosis, cardiovascular system diseases, digestive system diseases, and urinary system diseases. In addition, it reduces the quality of life and causes great harm to women's physical and mental health [3–5]. Science magazine even speculates that ovarian aging is the pacemaker of female collective aging and the initiating factor of multiorgan aging [6].

At present, the conventional treatment methods for ovarian aging mainly include the following: hormone replacement therapy; drug intervention; assisted reproductive technology; and treatment for perimenopausal symptoms caused by ovarian aging. However, these methods have various problems, such as increasing the risk of recurrence of cancer in cancer survivors, limited efficacy, large individual differences, and risk and unknown duration of treatment effects [7–9].

Mesenchymal stem cells (MSCs), a group of adult pluripotent stem cells with the ability to self-renew and differentiate, were originally discovered in bone marrow by Friedenstein, who described them as fibroblast-like cells present in vertebrate bone marrow. To date, MSCs can be obtained from almost all human tissues. In recent years, MSCs have become a research area of high activity due to their wide range of sources, low immunogenicity, strong immunomodulatory ability, and multidifferentiation potential, which can play a role in the repair and regeneration of tissue damage and degeneration in various ways, such as through direct participation and secretion of cytokines and exosomes [10–14]. In the treatment of ovarian senescence, MSCs from different tissue sources have shown sufficient advantages [15–17]. Our research group evaluated the role and mechanism of umbilical cord-derived MSCs after repeatedly administering MSCs to aging macaques and mice and confirmed that MSCs can reduce the expression levels of aging- and apoptosis-related genes and anti-inflammatory and immune regulation factors by reducing the amount of adipose tissue. In addition, ovarian aging can be improved by upregulating the P53 signaling pathway [18].

In our previous study, we identified and isolated a unique subgroup of MSCS, tentatively named highly active MSCS (HA-MSC), which are short rod-shaped fusiform or triangular cells that highly express SOX2, Nanog, and OCT4, the signature antigens associated with embryonic stem cells [19]. HA-MSCs have significantly higher proliferative ability and pluripotency than MSCs and can differentiate into neural, myocardial, and hepatoenteric progenitor cells from three dermal cell types in vitro, indicating that these cells are subomnipotent stem cells. Moreover, many experiments by our group have demonstrated that HA-MSCs have stronger resistance to ovarian senescence than MSCs. They can even partially restore the reproductive function of old macaques. The experiment has been completed, and the paper is being revised and reorganized.

To further explore the cellular and molecular mechanisms of HA-MSCs in the treatment of ovarian senescence and to provide more evidence supporting the efficacy of HA-MSCs, this experimental study used nonhuman primate macaques as the research subjects and screened naturally senescent female macaques to receive HA-MSC treatment through single nuclear transcriptome sequencing to explore the therapeutic effect of HA-MSCs in the treatment of ovarian senescence at the molecular level and to provide a reference scheme for the cellular and molecular mechanism of HA-MSC products in the treatment of ovarian senescence, in hopes of providing a new option for the stem cell treatment of ovarian senescence.

## Materials and methods

### Experimental materials

#### Animal origin

In this study, rhesus monkeys were purchased from the Laboratory Animal Center of Kunming Institute of Animal Sciences, Chinese Academy of Sciences. The experimental animal production license number was SCXK (Yunnan) K2017-0003. The young male rhesus monkey was 8 years old and weighed 2 kg. Female macaques were 22–28 years old and weighed 3.5–6.5 kg.

The animals were reared in the Laboratory Animal Center of Basic Medical Laboratory, 920th Hospital of the Joint Logistic Support Force of the Chinese People’s Liberation Army. The experimental animals, license No. SYXK (Military) 2017–0051, were reared in an ordinary clean environment of a normal size. All animal studies were approved by the Experimental Animal Ethics Committee of the 920th Hospital of the Joint Logistic Support Force of the People's Liberation Army. The approval number is 2020-034(Section)-01. Experimental operators have passed the professional training of laboratory animal practitioners.

**Fig. 1:**
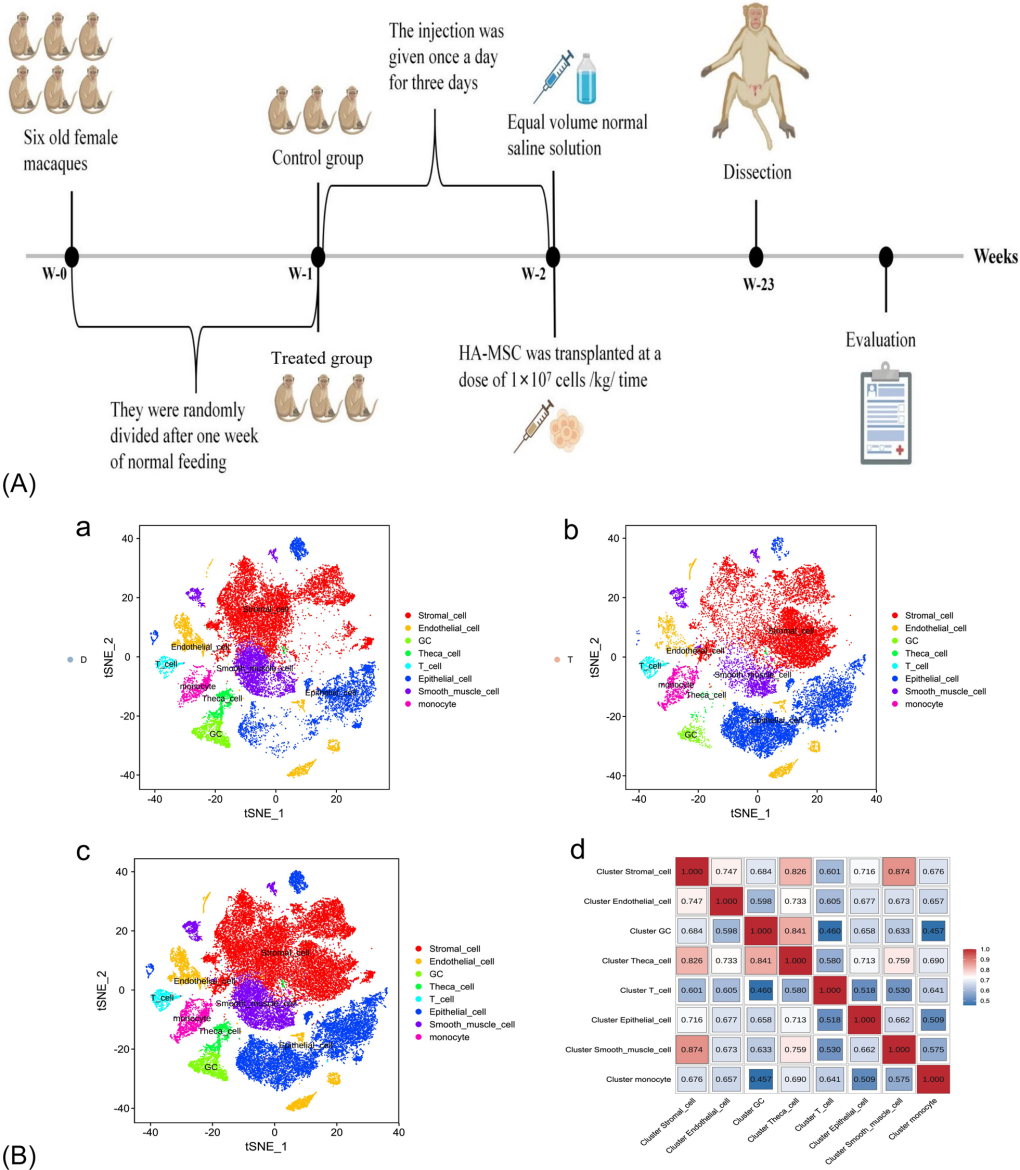
**A** Experimental flowchart. **B** Visualization of single-cell subpopulation classification. a tSNE diagram of single-cell subpopulation classification in the aging model group (Group D). b Single-cell subgroup classification t-SNE of the HA-MSC-treated group (T group). c Visual tSNE for subgroup classification of all groups. d Correlation heatmap between subgroups. When two cell subsets are highly correlated, they also have relatively similar gene expression patterns and may be the same cell type, which can provide certain guidance for the identification of cell subsets

HA-MSCs were diluted to 4 × 10^6^ cells/ml, and the treated group was transgrafted with HA-MSCs through the posterior vein at a dose of 1 × 10^7^ cells/kg, while the elderly model group was transfused with an equal volume of normal saline as the control once a day for three consecutive transfusions. At the end of the 5-month observation period, rhesus monkeys were anesthetized intravenously with excess 3% sodium pentobarbital. After the monkeys entered unconsciousness, the femoral artery was quickly bled, and the animals were euthanized [20, 21]. Once the abdominal cavity was exposed, the ovaries were located, isolated, and sent to Gidio for 10X Genomics single-cell transcriptome sequencing. The experimental flowchart is shown in Fig. 1A.

#### Sources of HA-MSCs and human ovarian granulosa cells (hGCs)

Human HA-MSCs were provided by our laboratory. Briefly, HA-MSC complete medium was prepared, 20 ml of TeSR-E8 supplement (purchased from STEMCELL) was added to 480 ml of TeSR-E8 basal medium, mixed upside-down, and 20 ml of CloneR (purchased from STEMCELL) was added. A pH range of 7.2–7.4 adjusted by 6.6% NaHCO3 was used for HA-MSC culture. Dilute laminin LN521 stock solution (purchased from Biolamina) with 1 × DPBS (Ca2 + /Mg2 +) to 5 μg/ml, add 5 ml of coating solution per T75 cm2, evenly cover the whole bottom of the bottle, incubate at 2–8 °C overnight, and check the next day. Conventionally cultured human MSCs (obtained from human umbilical cord culture) were inoculated at a density of 5000 cells/cm^2^ into a new culture vial coated with LN521 protein. HA-MSCs were obtained by expanding the culture in an incubator at 37 °C and 5% CO2. Purchased hGC strains were obtained from Wuhan Punosai Life Technology Co., Ltd.

### Experimental methods

#### Single nuclear transcriptome sequencing at 10X Genomics

After the macaques were euthanized, the ovarian tissues were collected, labeled, and placed in a frozen tube. The tubes were frozen in liquid nitrogen and stored at − 80 °C for 24 h. After labeling, the tissue was removed and sent to Guangzhou Gidio Biotechnology Co., Ltd. for 10X Genomics single nuclear transcriptomics sequencing under the condition of sample delivery. There were six ovaries used for sequencing in this study. The ovaries were divided into two groups: three in the aging model group and three in the HA-MSC-treated group. However, one sample in each of the two groups failed the quality inspection due to possible problems in the sampling process or transportation process. There were four final sequencing samples that were split into two groups: two in the aging model group and two in the HA-MSC-treated group.

#### Single nuclear transcriptome sequencing analysis of 10X Genomics

1. There were some residual abnormal cells in cellranger cell filtration after automatic recognition based on gene expression. Therefore, before classifying subgroups, we further filtered the residual abnormal cells. The process is divided into two steps: The first step is to remove polycells based on the polycell rate; the second step is to remove abnormal cells based on the gene expression, UMI number, and mitochondrial gene ratio.
2. After removing low-quality cells, we used harmony for data consolidation and batch effect correction.
3. To understand the molecular expression characteristics of each cell subpopulation, we screened the upregulated genes of each cell subpopulation.
4. Seurat software was used to analyze the differences between groups based on the subgroup information and sample information of cells.
5. Gene Ontology (GO) is an internationally standardized gene function classification system that provides a set of dynamically updated standard vocabularies to comprehensively describe the properties of genes and their gene products in organisms. First, the differentially expressed genes were mapped in terms of the GO database. Then, the number of differentially expressed genes in each term was calculated to count the number of differentially expressed genes in the list with GO functions. Finally, a hypergeometric test was used to determine the GO entries of differentially expressed genes compared with the background.
6. In organisms, different genes coordinate with each other to perform their biological functions, and pathway-based analysis is helpful to further understand the biological functions of genes. The main public database for pathways is the Kyoto Encyclopedia of Genes and Genomes (KEGG). As the unit of significant pathway enrichment analysis, KEGG was used to determine the significantly enriched pathways of different genes compared with the whole background through a hypergeometric test, and then, the most important signal transduction pathways and biochemical metabolic pathways represented by differentially expressed genes were determined through significant enrichment screening.
7. Pseudotemporal analysis, also known as cell locus analysis, was performed using the Monocle tool. Based on the expression patterns of key genes, Monocle uses a pseudotemporal approach to sequence single cells. The gene expression matrix generated by 10X Genomics was imported into Monocle to construct a cell differentiation locus, and cell trajectory visualization was performed for different differentiation states (states), different samples, and different cell subgroups (clusters).
8. CellPhoneDB software uses a single-cell gene expression matrix to analyze the number of ligand–receptor pairs in cell pairs and their expression information, construct a cell interaction network diagram, and predict the potential communication relationship between cells.

#### Establishment of the hGC aging model

Human hGC strains were inoculated into 6-well culture plates, and when the cell confluence reached 70–80%, 100, 200, 300, 400, and 500 μmol/L H_2_O_2_ was added to hGCs for 24, 48, and 72 h, respectively. According to the growth morphology, proliferation, and cell cycle of hGC, the suitable induction conditions of the hGC senescence model were analyzed.

#### HA-MSCs cocultured with aging hGCs and q-PCR verification

The proliferation and growth morphology of aging hGCs were observed under an optical microscope after coculture with P3 generation HA-MSCs for 48 h. The ultrastructures of hGCs were observed under a transmission electron microscope. β-galactosidase staining was performed to observe the changes in hGC β-galactosidase gene expression. The expression levels of the hGC P16 and P21 genes were detected by immunohistochemical staining. The P16 and P21 antibodies were purchased from Proteintech. The migration ability of cocultured cells was detected by a cell migration assay. The genes screened by single-nucleus transcriptome sequencing were verified in vitro by q-PCR. Briefly, hGCs from the coculture group and the aging group were collected, RNA was extracted, and reverse transcription and quantitative PCR were performed to detect the expression of the IGF2, RAF1, MAP2K1, and RPS6kA genes. The primers are shown in Table 1.

**Table 1:** Primers for quantitative PCR

Target gene	Primer sequence
RPS6kA F	acc ttt tca ctc ggc ttt cc
RPS6kA R	att ccc agg ctg tgc aaa tg
MAP2K1 F	tca tct gga gat caa acc cgc aat c
MAP2K1 R	cca tcg ctg tag aac gca cca tag
RAF1 F	caa aga gag cgg gca cca gta tc
RAF1 R	aga gcc tga ccc aat ccg agt g
ERBB4 F	agc acc caa tca agc tca ac
ERBB4 R	aac cgt tcc aaa agc acc tg
IGF2 F	ccg tgg cat cgt tga gga gtg
IGF2 R	acg ggg tat ctg ggg aag ttg tc

### Statistical analysis

The statistical methods used on the data obtained in this experiment were as follows: All measurement data were expressed as the mean ± standard deviation ($\overline{x} \pm s$), and t-tests and one-way ANOVAs were used for the samples of two group or three group comparisons using SPSS statistical analysis software. The p value was used to determine whether there was statistical significance, and *p* < 0.05 was considered a significant difference (*p < 0.05, **p < 0.01, and ***p < 0.001). Otherwise, the difference was not statistically significant.

## Results

### Single nuclear transcriptome sequencing analysis

#### Single-cell subpopulation classification

By classifying the two groups of cells by single-cell subsets, the differences in each group of cell subsets can be intuitively seen, and the cytological mechanism of HA-MSC therapy can be analyzed.

The aging model group was named group D, and the HA-MSC-treated group was named group T. Through single-cell RNA-seq analysis of the macaque ovary, different ovarian cell subsets with varying transcriptional characteristics were determined. The correlation between the two cell subsets was calculated, and a heatmap was drawn in which the two highly correlated cell subsets had relatively similar gene expression patterns. These may be the same cell type, providing some guidance for the identification of artificial cell subpopulations (Fig. 1B). Finally, we identified eight cell clusters: stromal cells, endothelial cells, granular cells, theca cells, T cells, epithelial cells, smooth muscle cells, and monocytes.

#### Analysis of upregulated genes

By analyzing the upregulated genes, we can intuitively see which cells are mainly distributed by the expression levels of these genes and which cells are mainly acted on during HA-MSC therapy.

**Fig. 2:**
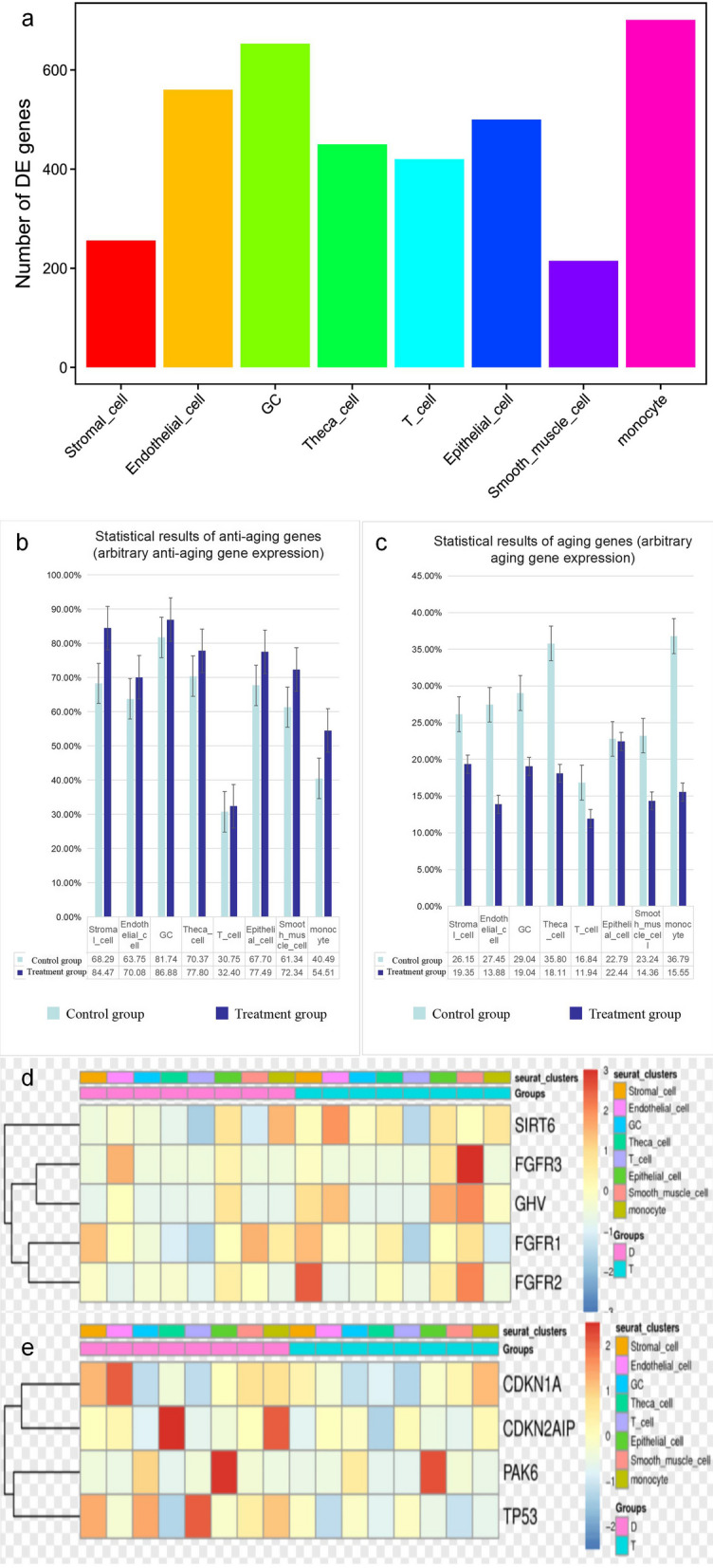
Analysis of upregulated genes. **a** Statistical map of the number of upregulated genes in each subpopulation (horizontal coordinate is subpopulation, and vertical coordinate is number of upregulated genes), 256, 560, 653, 450, 420, 500, 215, and 701. **b** Statistics showed that the proportion of anti-aging genes expressed in the HA-MSC-treated group was higher than that in the aging model group. **c** Statistics showed that the proportion of age-related genes expressed in the aging model group was higher than that in the HA-MSC-treated group. **d** Expression calorigram of five anti-aging genes before and after treatment (within each group, cells of the same cell subgroup are classified together, and the subgroup of each cell is marked with seurat_cluster above the heatmap; the darker the color of the small grid indicates higher gene expression). **e** Expression calorigrams of 4 aging genes before and after treatment

The results showed that there were 3755 upregulated genes, mainly distributed in endothelial cells (560), granulosa cells (653), and monocytes (701) (Fig. 2). It was speculated that treatment of senescent ovaries with HA-MSCs mainly regulated these three kinds of cells. The proportion and expression of anti-aging genes were higher in the HA-MSC-treated group, while the proportion and expression of age-related genes were lower in the HA-MSC-treated group.

#### Differentially expressed gene analysis

Differentially expressed gene analysis can determine which genes have altered expression in the treated group and which genes have altered expression in the aging group.

**Fig. 3:**
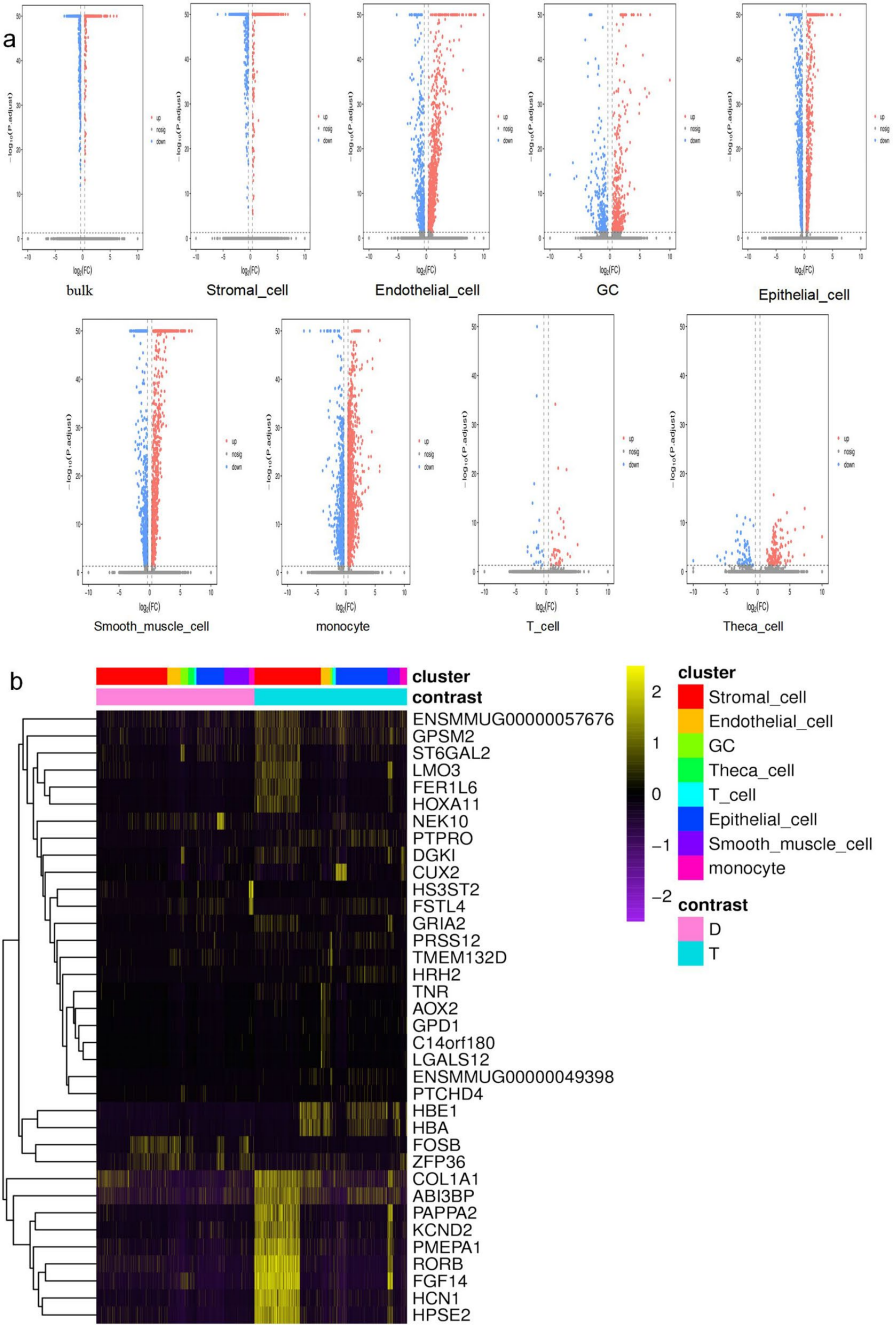
Differentially expressed gene analysis. **a** The thermal map of enrichment scores of each group in each subgroup. The *x*-coordinate represents the difference multiple pairs between the two groups, and the *y*-coordinate represents the difference FDR (Q value)-Log10 value between the two groups. Red was used for expression level upregulation, and blue was used for expression level downregulation. Red and blue dots indicate differences (FDR < 0.05 and more than twofold difference were used as the judging criteria); gray dots indicate no difference in gene expression level. **b** Differential gene expression heatmap: Each row represents a gene, and each column represents a cell. Cells in the same group in the figure are grouped together, and the group of each cell is marked with contrast above the heatmap. Within each group, cells of the same cell subgroup are grouped together, and each cell belongs to a cell subgroup labeled as a cluster above the heatmap

The differences in gene expression were obvious in stromal cells, endothelial cells, GCs, epithelial cells, smooth muscle cells, and monocytes (Fig. 3) but not in theca cells and T cells. Among the genes with significant differences, the genes that were highly expressed after HA-MSC treatment were mainly those related to growth factors, protein synthesis regulation, and growth and development, such as GPSM, PAPPA2, and FGF. However, in the aging model group, the genes that were highly expressed were mainly FOSB, HS3ST2, and FSTL4, which are related to tumors, cancer, and other diseases.

#### GO enrichment analysis

GO enrichment analysis can reveal which functions the differentially expressed genes are mainly enriched in.

**Fig. 4:**
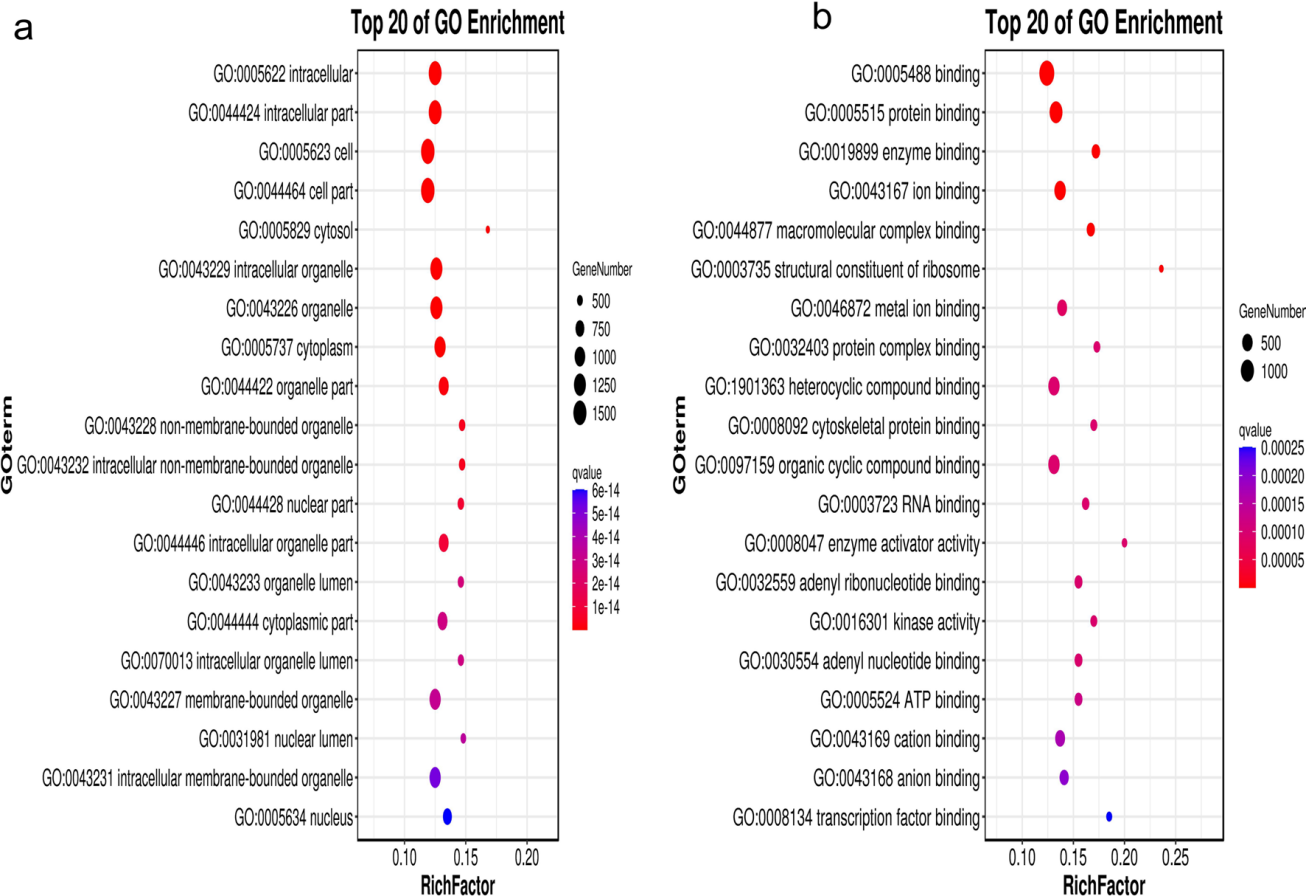
GO enrichment analysis. GO enrichment bubble diagram (vertical coordinate is GO term, horizontal coordinate is enrichment factor, that is, divide the differentially expressed genes in the GO term by all the quantities, the size represents the quantity, the redder the color, the smaller the Q value). **a** Go-enriched bubble diagram of a differential gene on molecular function. **b** Go-enriched bubble diagram of differential gene on cell component

GO annotation analysis showed that differentially expressed genes were mainly enriched in cell components and molecular functions (Fig. 4). Analysis of the first 20 GO terms with the lowest Q value showed that differentially expressed genes enriched in cell components mainly affected organelles. The differentially expressed genes enriched in molecular function mainly affected the binding effect, including the binding of enzymes, ions, proteins, nucleic acids, and various compounds.

#### KEGG analysis

KEGG analysis showed that the differentially expressed genes were mainly concentrated in specific pathways.

**Fig. 5:**
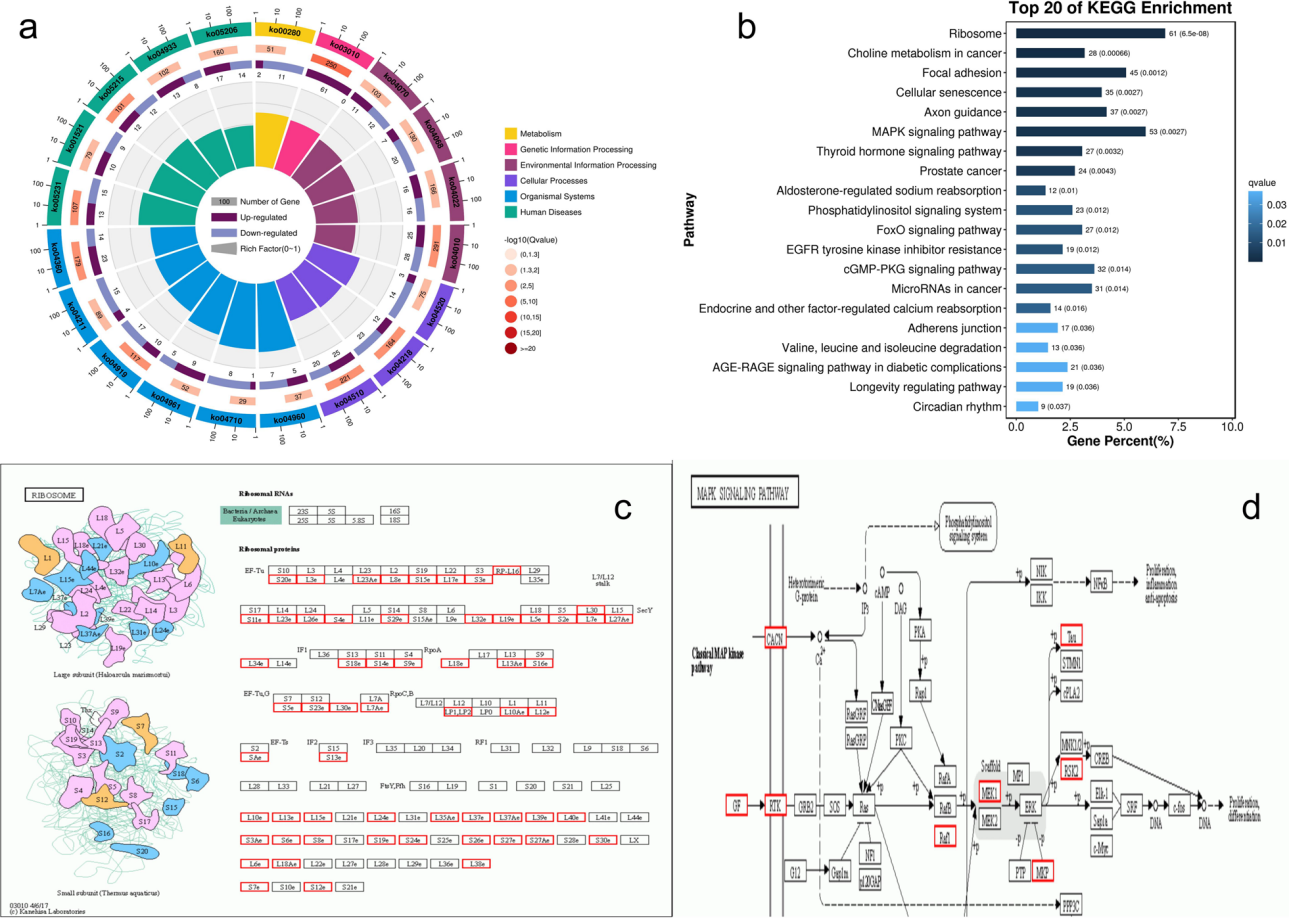
KEGG enrichment analysis. **a** KEGG enrichment circle diagram (The first circle: the top 20 enriched signaling pathways, the outer circle is the coordinate scale of gene number, and different colors represent different A class; Circle 2: the number and Q values of this pathway in background genes. The more genes there are, the longer the bars, the smaller the Q values, and the redder the colors. The third circle: upregulated gene ratio map, dark purple represents upregulated genes, light purple represents downregulated genes, and specific values are displayed at the bottom; and Circle 4: enrichment values of each pathway, i.e., the number of differentially expressed genes in the pathway divided by all the numbers (each grid line in the background represents 0.1). **b** KEGG enrichment bar graph (ordinate is pathway, and abscis is the percentage of the number of pathways in the number of differentially expressed genes; the smaller the Q value, the darker the color; and the number of pathways and Q value are shown in the column). **c** Ribosome pathway (red for upregulation). **d** MAPK signaling pathway

Significant enrichment of pathways showed that differentially expressed genes were significantly enriched in cellular senescence, the MAPK signaling pathway, the FoxO signaling pathway, and other pathways (Fig. 5). Among these, the ribosome pathway had the highest degree of differentially expressed gene enrichment, and all the changes in large and small subunits were upregulated. The MAPK signaling pathway runs through other significantly enriched pathways.

#### Quasitime analysis

The quasitime analysis showed that GC cells were in different developmental stages in the two groups.

**Fig. 6:**
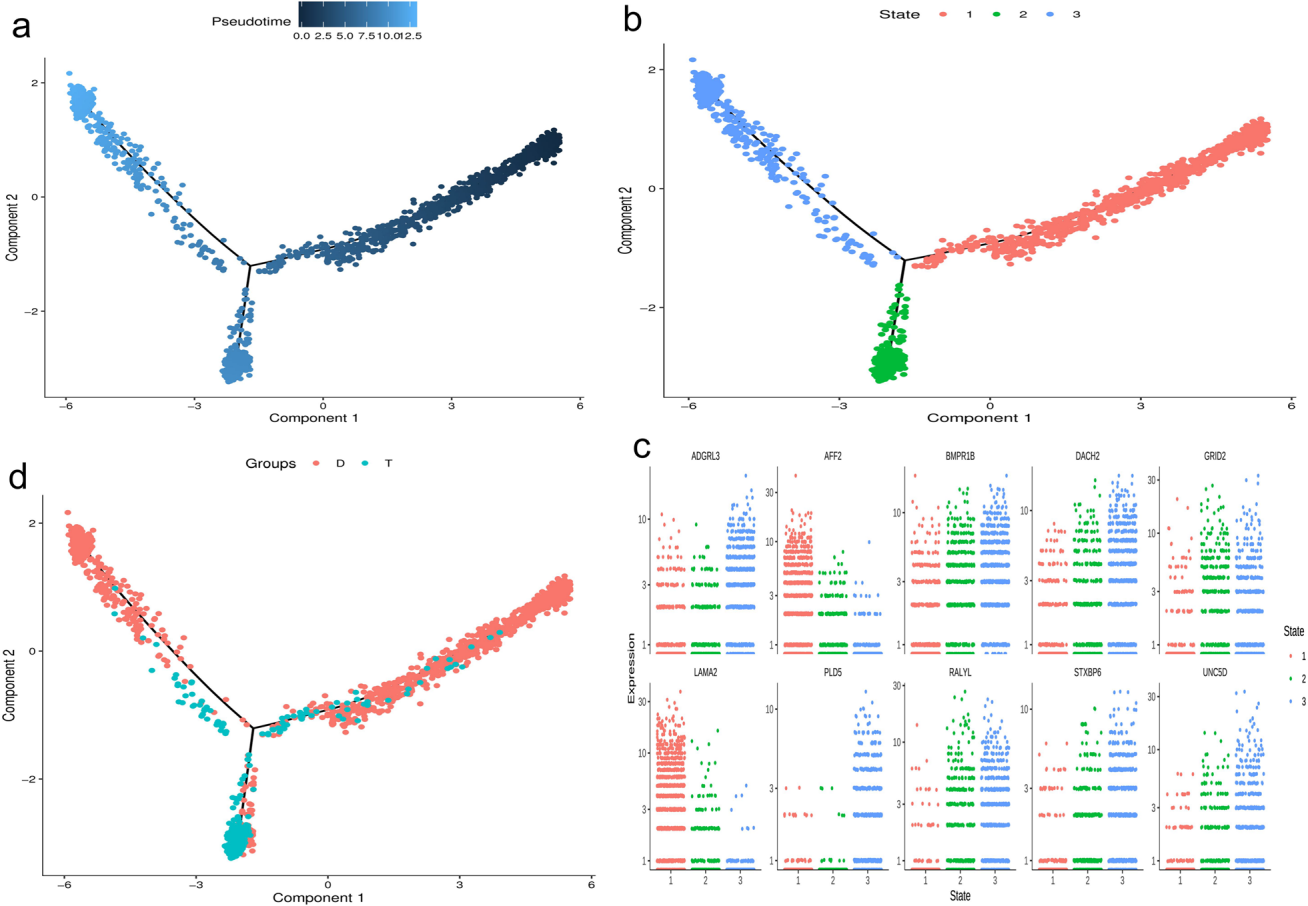
Quasitemporal analysis. **a** Quasitemporal distribution diagram of cell tracks (different points represent different cells; the darker the point color is, the smaller the pseudotime, and the earlier the development stage). **b** Cell locus visualization (the distribution of cells in different stages of differentiation within the cell locus). **c** Cell track visualization (distribution of cells in different groups within a cell track). **d** Scatter plots of differentially expressed genes in different differentiation stages (showing the top 10 genes)

According to the time-like analysis, GC cells in the HA-MSC-treated group and the aging model group were in different developmental stages, and almost all GC cells in the aging model group were in the period of regeneration but nondivision, proliferation, and apoptosis, while almost all GC cells in the HA-MSC-treated group were in the state of division and differentiation (Fig. 6).

#### Cell interaction analysis

Cell interaction analysis compares the number of cell receptor pairs between the two groups.

**Fig. 7:**
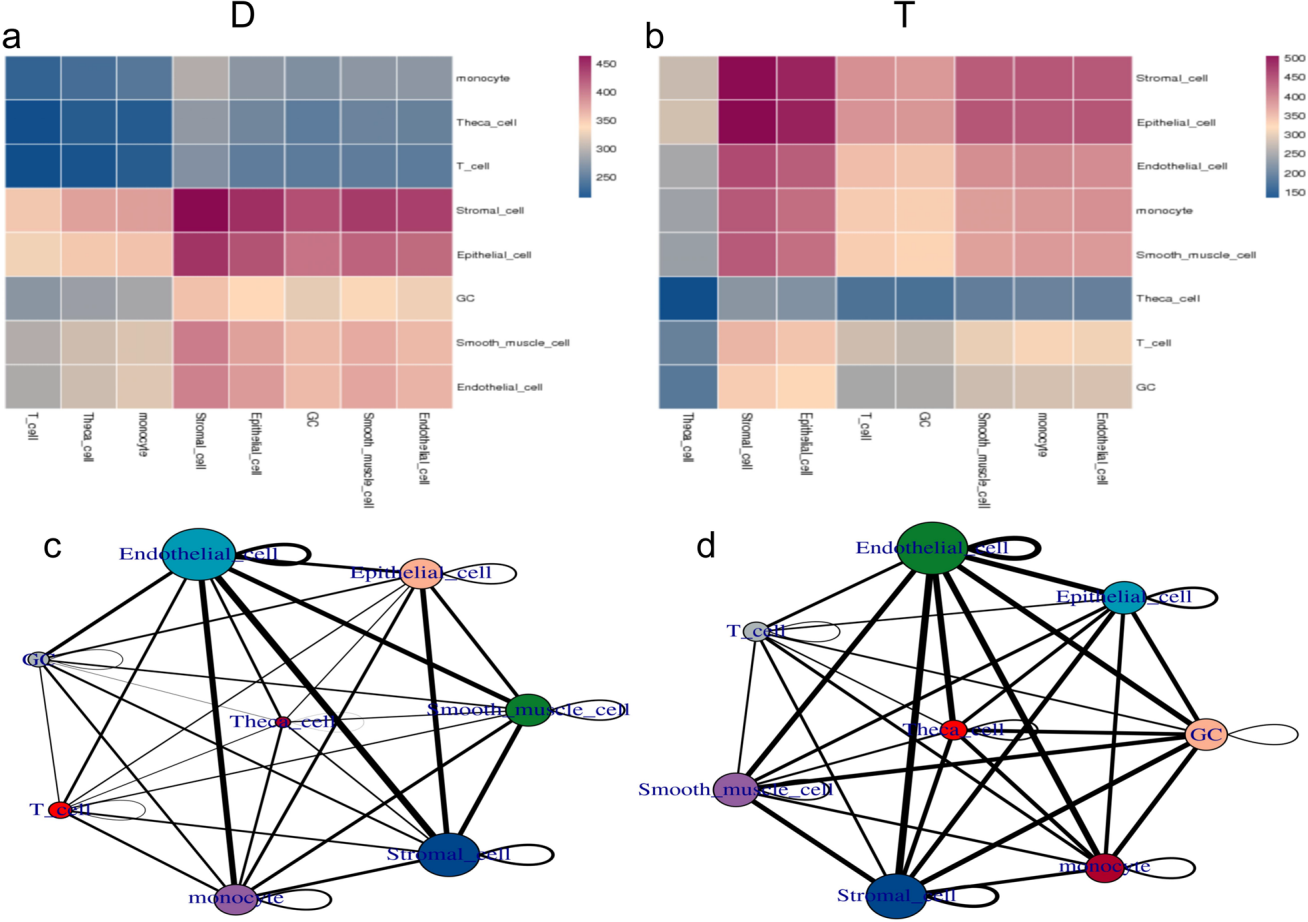
Cell interaction analysis. **a** Heatmap of the number of ligand–receptor pairs possessed by each pair of cells (horizontal is ligand cell subgroup, vertical is receptor cell subgroup, and different colors are used to represent the number of ligand–receptor pairs possessed by each pair of cells. The smaller the number of ligand–receptor pairs possessed by this pair of cells, the bluer the color of the cell, and the more ligand–receptor pairs possessed by this pair, the redder the color of the cell). **b** Cell interaction network diagram, bubbles represent cell subsets, and the size of bubbles is determined by the number of significantly enriched receptor pairs between subsets and all interacting subsets. The larger the bubbles are, the larger the total number, indicating a stronger association of subsets in the population. The number of significantly enriched receptor pairs between subgroups is represented by a line. The number of significantly enriched receptor pairs between subgroups determines the thickness of the line. The thicker the line is, the greater the number of significantly enriched receptor pairs between subgroups, and the stronger the communication relationship between subgroups. **c** Cell interaction network diagram of the aging group. **d** Cell interaction network diagram of the treatment group

The analysis of intercellular communication showed that compared with the aging model group, the number of cell receptor pairs in each subgroup of the HA-MSC-treated group was increased overall (Fig. 7).

### Establishment of the hGC aging model

#### Effects of different concentrations of H_2_O_2_ on hGCs

By establishing the hGC senescence model, the cellular and molecular mechanisms of HA-MSCs on senescent hGCs can be observed.

**Fig. 8:**
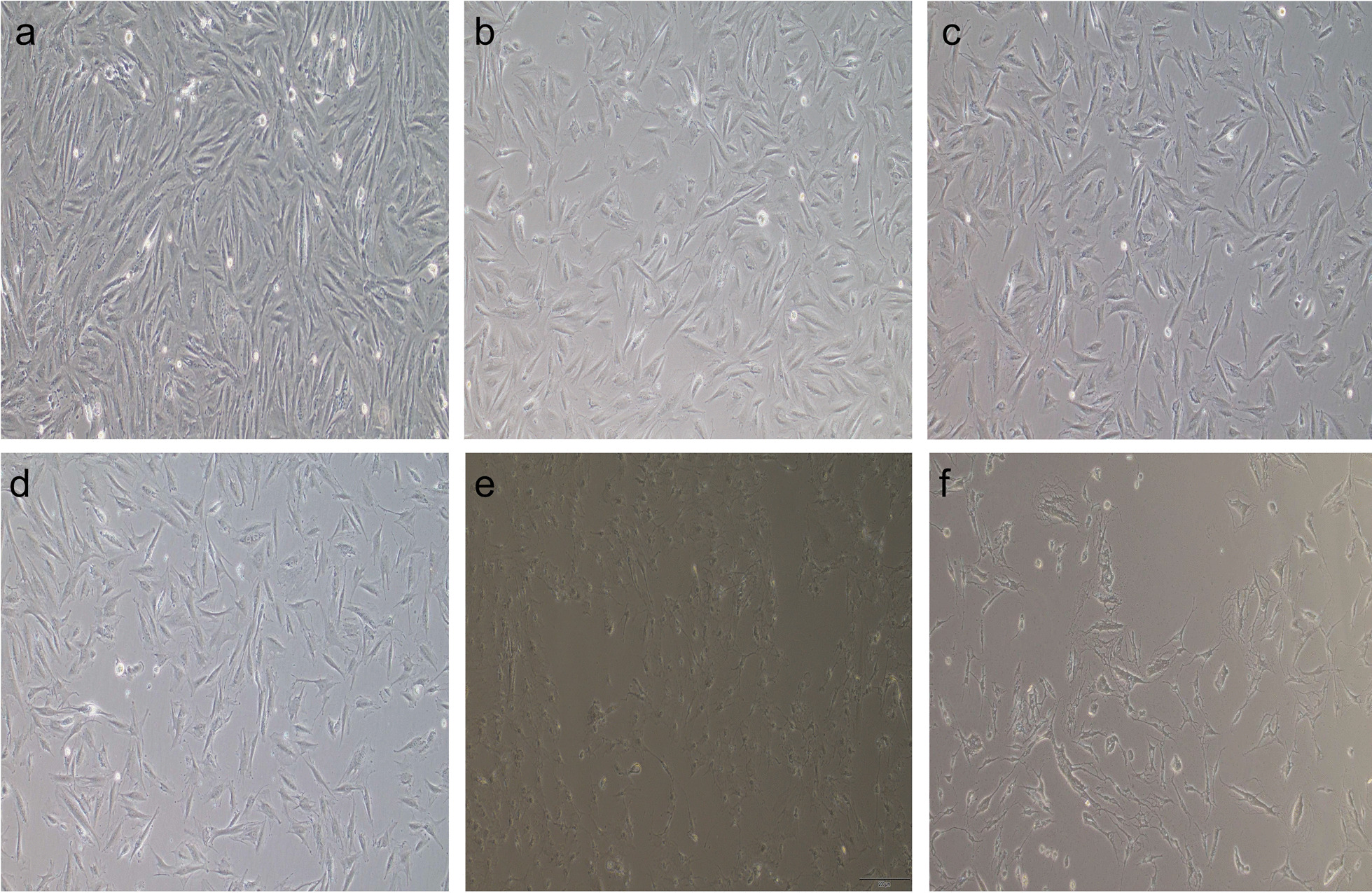
Morphological observation of hGCs under different concentrations of H_2_O_2_ (100 ×). **a** P4 generation hGCs; **b** hGCs treated with 100 μmol/L H_2_O_2_; **c** hGCs treated with 200 μmol/L H_2_O_2_; **d** hGCs treated with 300 μmol/L H_2_O_2_; and **e** hGCs treated with 400 μmol/L H_2_O_2_. **f** hGCs were treated with 500 μmol/L H_2_O_2_

The growth of hGCs in 100 μmol/L H_2_O_2_ coculture was not significantly different from that in the normal control group. At 200 μmol/L and 300 μmol/L H_2_O_2_ treatment, the proliferation ability of hGCs was significantly reduced, and the cells were flattened and enlarged compared with the normal control group. Almost all hGCs treated with 400 and 500 μmol/L H_2_O_2_ died (Fig. 8).

#### Effects of H_2_O_2_ on the hGC cell cycle at 200 μmol/L and 300 μmol/L

By observing the cell cycle of hGCs under different concentrations of hydrogen peroxide, we found the optimal concentration of hydrogen peroxide to induce hGC senescence.

**Fig. 9:**
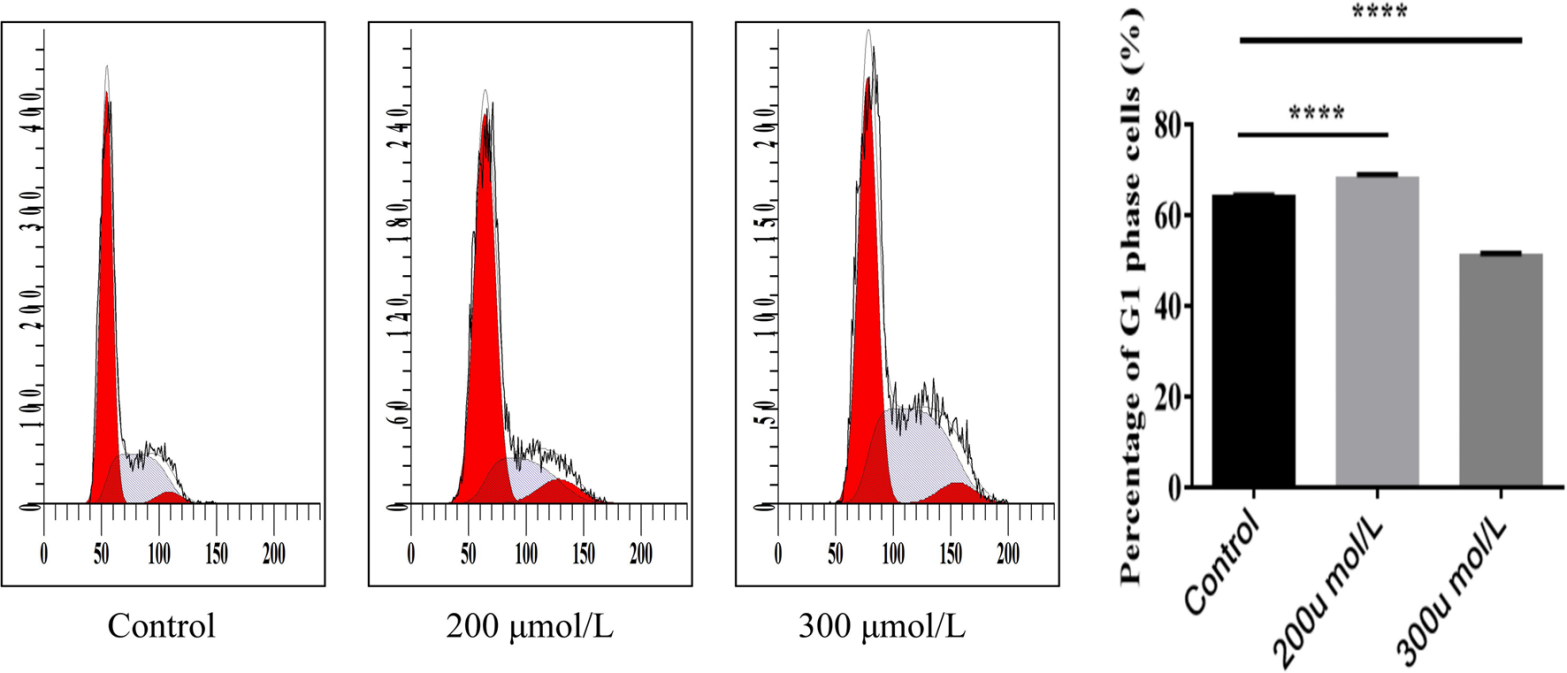
hGC cell cycle was determined by flow cytometry. The proportion of hGCs in G0/G1 phase was the highest in the 200 μmol/L H_2_O_2_ treatment (*p* < 0.01)

Flow cytometry analysis (Fig. 9) showed that the proportion of hGCs in the G0/G1 phase was the highest after treatment with 200 μmol/L H2O2 (*p* < 0.01).

#### Senescence of hGCs treated with 200 μmol/L H_2_O_2_ at different times

The effects of β-galactosidase staining with 200 μmol/L H_2_O_2_ at different times were observed to find the optimal staining times.

**Fig. 10:**
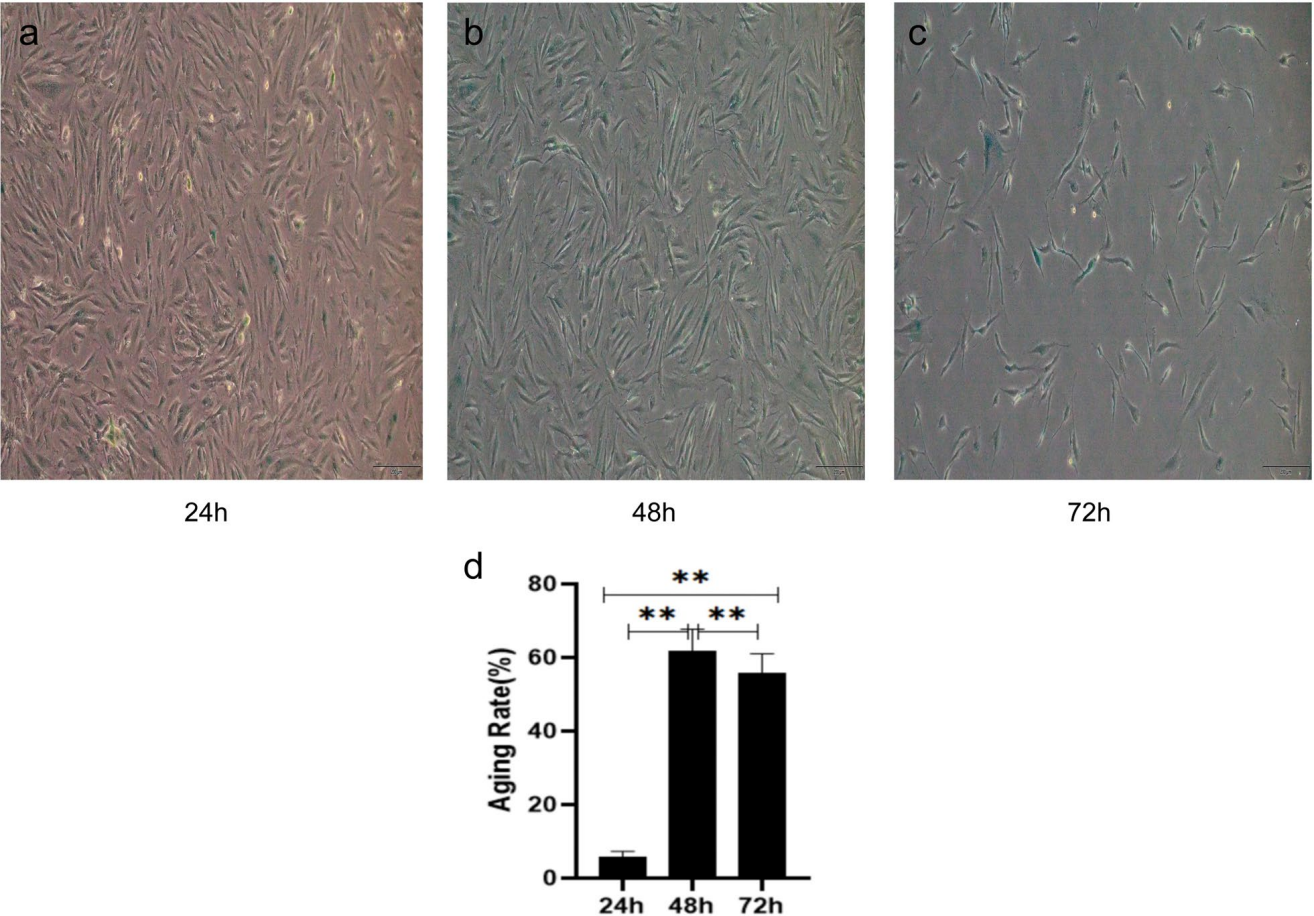
hGC β-galactosidase staining (100 ×). With the extension of H_2_O_2_ treatment time, the cells gradually became senescent, β-galactosidase activity gradually increased, and the blue staining rate gradually increased. Compared with 48 h, although the cells were more in line with the senescence condition at 72 h, the number of remaining cells was small compared with the 48-h condition. Therefore, 200 μmol/L H_2_O_2_ was selected to treat hGCs for 48 h for follow-up experiments. **a** β-galactosidase staining of hGC after 24h H_2_O_2_ treatment **b** β-galactosidase staining of hGC after 48h H_2_O_2_ treatment **c** β-galactosidase staining of hGC after 72h H_2_O_2_ treatment **d** Statistical graph of hGC senescence rate

β-galactosidase staining was performed at 24 h, 48 h, and 72 h in hGCs cocultured with 200 μmol/L H_2_O_2_, and the blue staining rate was the highest at 48 h (*p* < 0.01) (Fig. 10). Finally, treatment with 200 μmol/L H_2_O_2_ for 48 h was selected as the appropriate condition for the induction of hGC senescence, and the hGC senescence model was obtained by batch induction under these conditions for further experiments.

### Effect of HA-MSCs on aging hGCs

#### Effects of HA-MSCs on the morphology of aging hGCs

By observing the morphological changes in aging hGCs and HA-MSC coculture, it was found that HA-MSCs can promote the proliferation of hGCs.

**Fig. 11:**
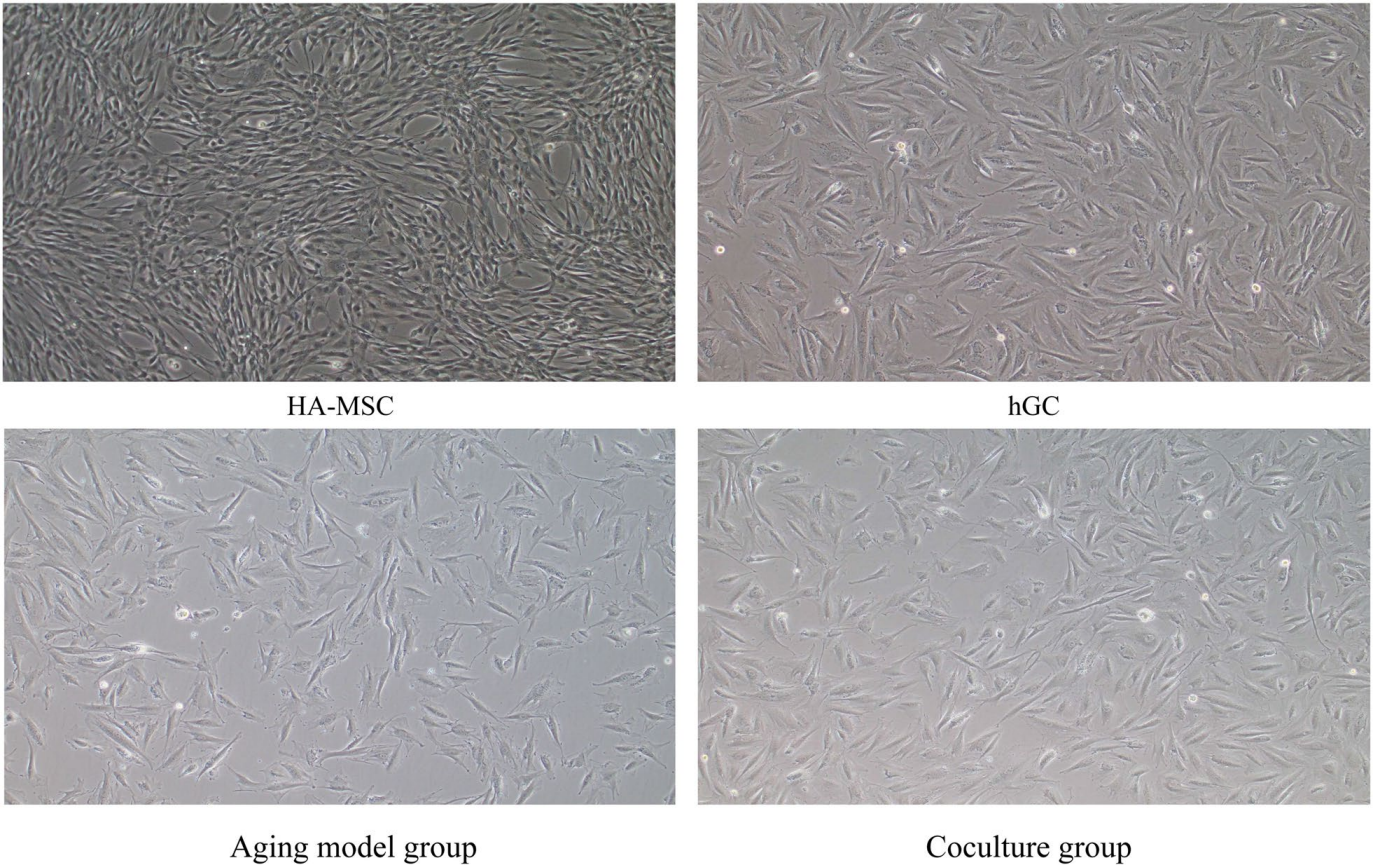
Morphological changes in hGCs after HA-MSC coculture (100 ×). After coculture, the cells showed a spindle shape or polygon shape, and the number of cells increased, indicating that the proliferation activity of aging hGCs was restored

Optical microscopy showed that the cells after coculture were spindle- or polygon-shaped, and the number of cells increased, indicating that the proliferation activity of aging hGCs was restored to some extent (Fig. 11).

#### Effects of HA-MSCs on the ultrastructure of hGCs

The morphological changes in aging hGCs and HA-MSC coculture were observed by electron microscopy, and it was found that hGCs were younger.

**Fig. 12:**
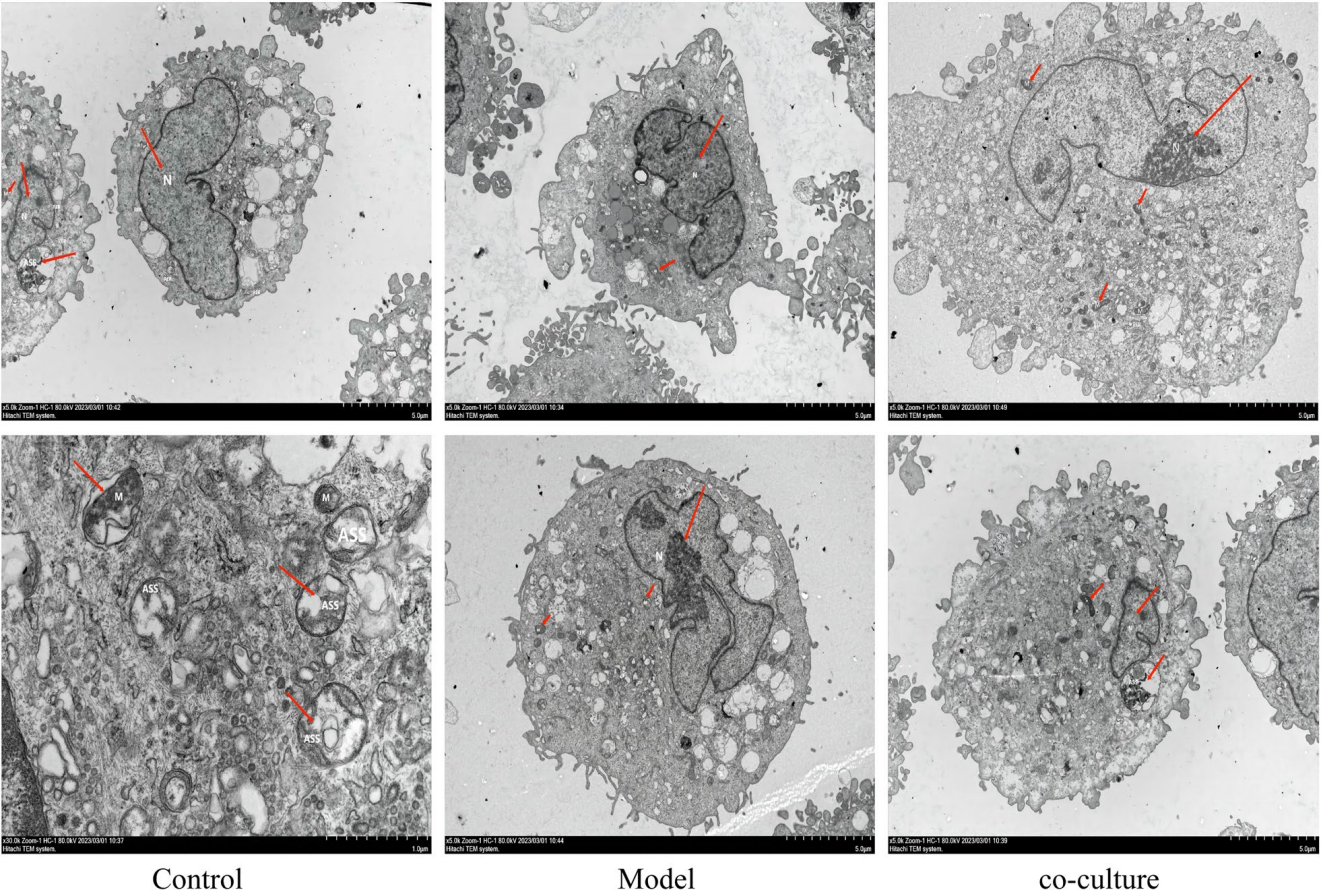
Ultrastructural changes in hGCs after HA-MSC coculture (1000 × and 5000 ×). Nucleus (N), mitochondria (M), and autolysosome (ALS). In cocultured hGCs, chromatin distribution was uniform, mitochondria were still swollen, rough endoplasmic reticulum was slightly expanded locally, and a few autophagosomes could be seen inside the cells

The results of transmission electron microscopy (Fig. 12) showed heterochromatin aggregation, patchy distribution, mitochondrial swelling, slight expansion of the endoplasmic reticulum, and no obvious autophagosomes. In cocultured hGCs, chromatin distribution was uniform, mitochondria were still swollen, rough endoplasmic reticulum was slightly expanded locally, and a few autophagosomes could be seen inside the cells.

#### Changes in β-galactosidase activity of HA-MSCs against hGCs

β-galactosidase staining showed that the percentage of senescent cells decreased after coculture of hGCs and HA-MSCs.

**Fig. 13:**
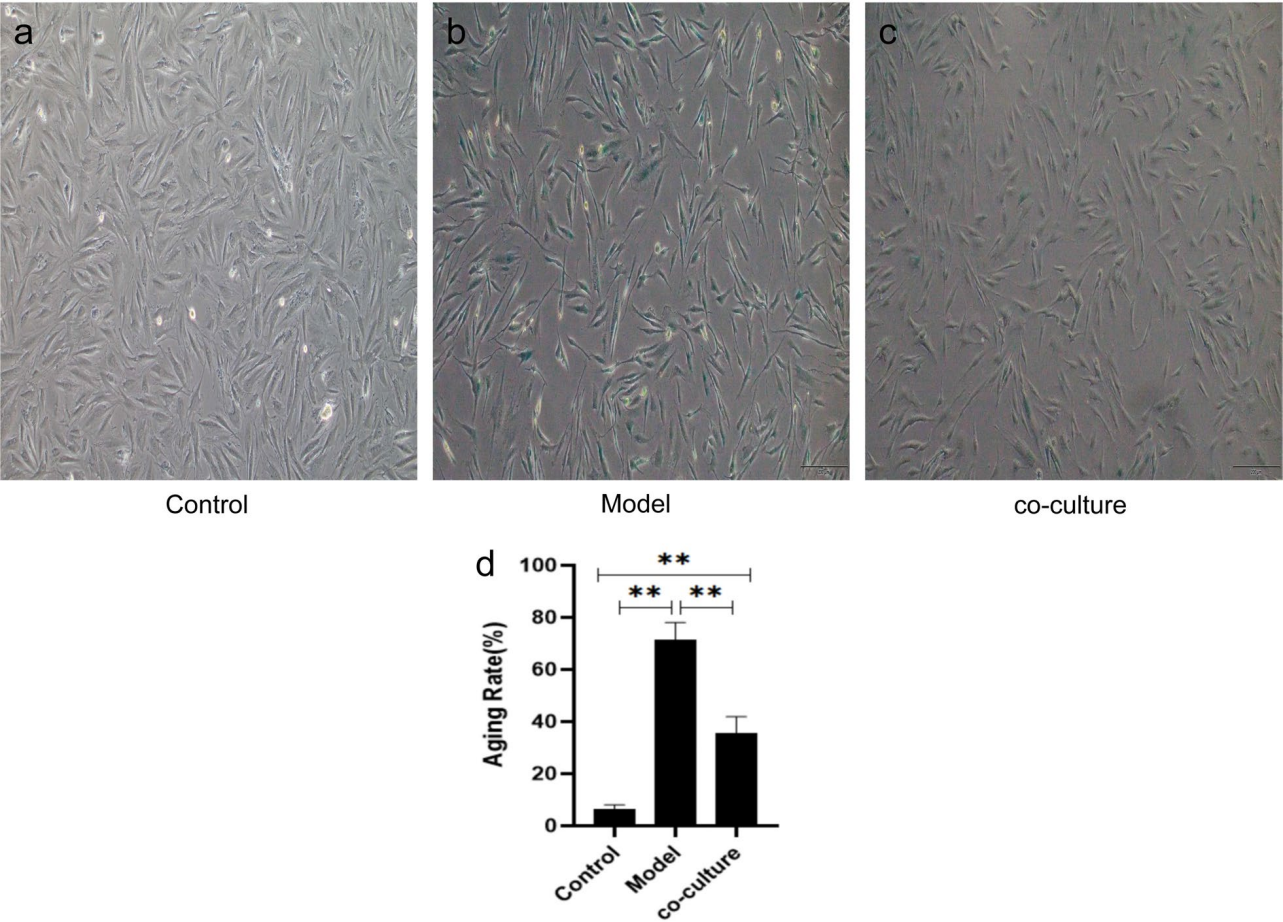
hGC β-galactosidase staining (100 ×). The results showed that the rate of cyanosis decreased after coculture (*p* < 0.01). **a** β-galactosidase staining of normal hGC; **b** β-galactosidase staining of senescent hGC; **c** β-galactosidase staining of hGC after HA-MSC co-culture; **d** Statistical graph of hGC senescence rate

β-galactosidase staining of the three states of hGCs showed that the blue staining rate decreased after coculture (*p* < 0.01) (Fig. 13).

#### The expression of hGC p16 and p21 was detected by immunohistochemical staining

**Fig. 14:**
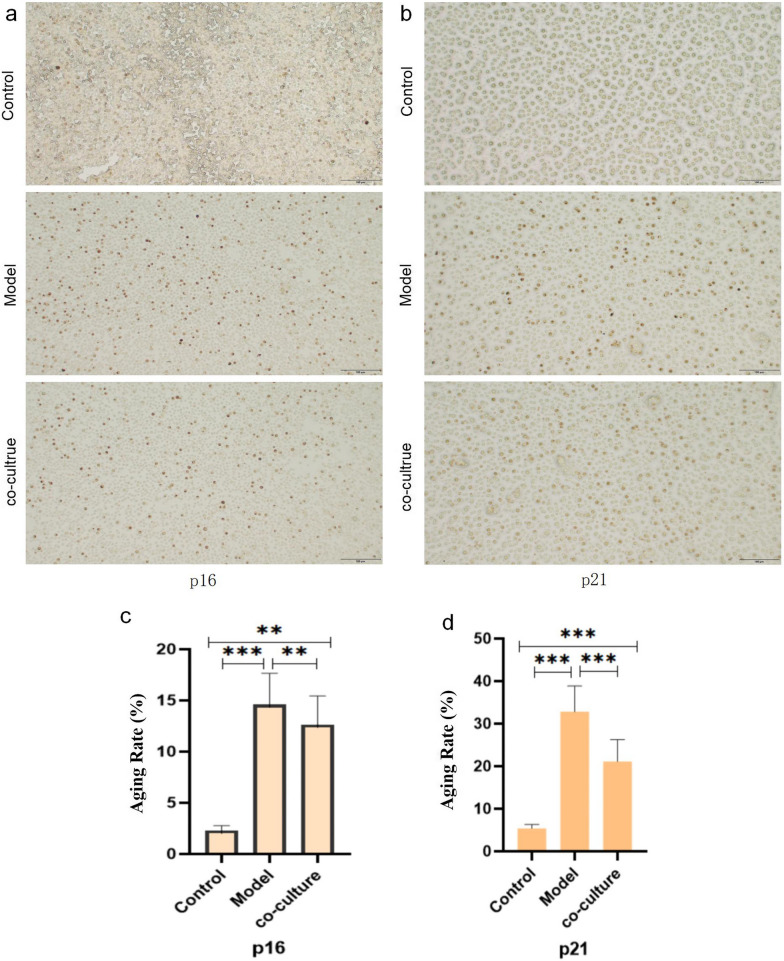
hGC immunohistochemical staining (200 ×). The results showed that the proportion of hGCs expressing p16 was 2.3 ± 0.48% in the control group, 14.62 ± 3.03% in the aging model group, and 12.65 ± 2.79% in the coculture group. The proportion of hGCs expressing p21 was 5.38 ± 0.93% in the control group, 32.8 ± 6.07% in the model group, and 21.1 ± 5.16% in the coculture group. **a** Immunohistochemical staining of p16 in hGC of three groups **b** Immunohistochemical staining of p21 in hGC of three groups **c** Statistical chart of positive rate of p16 **d** Statistical chart of positive rate of p21.

Immunohistochemical staining of p16 and p21 protein expression showed that the proportion of positive cells decreased after coculture (*p* < 0.01) (Fig. 14).

#### Cell migration ability was detected by cell migration assay

Immunohistochemistry revealed a decrease in P16- and P21-positive cells after coculture of aging hGCs and HA-MSCs.

**Fig. 15:**
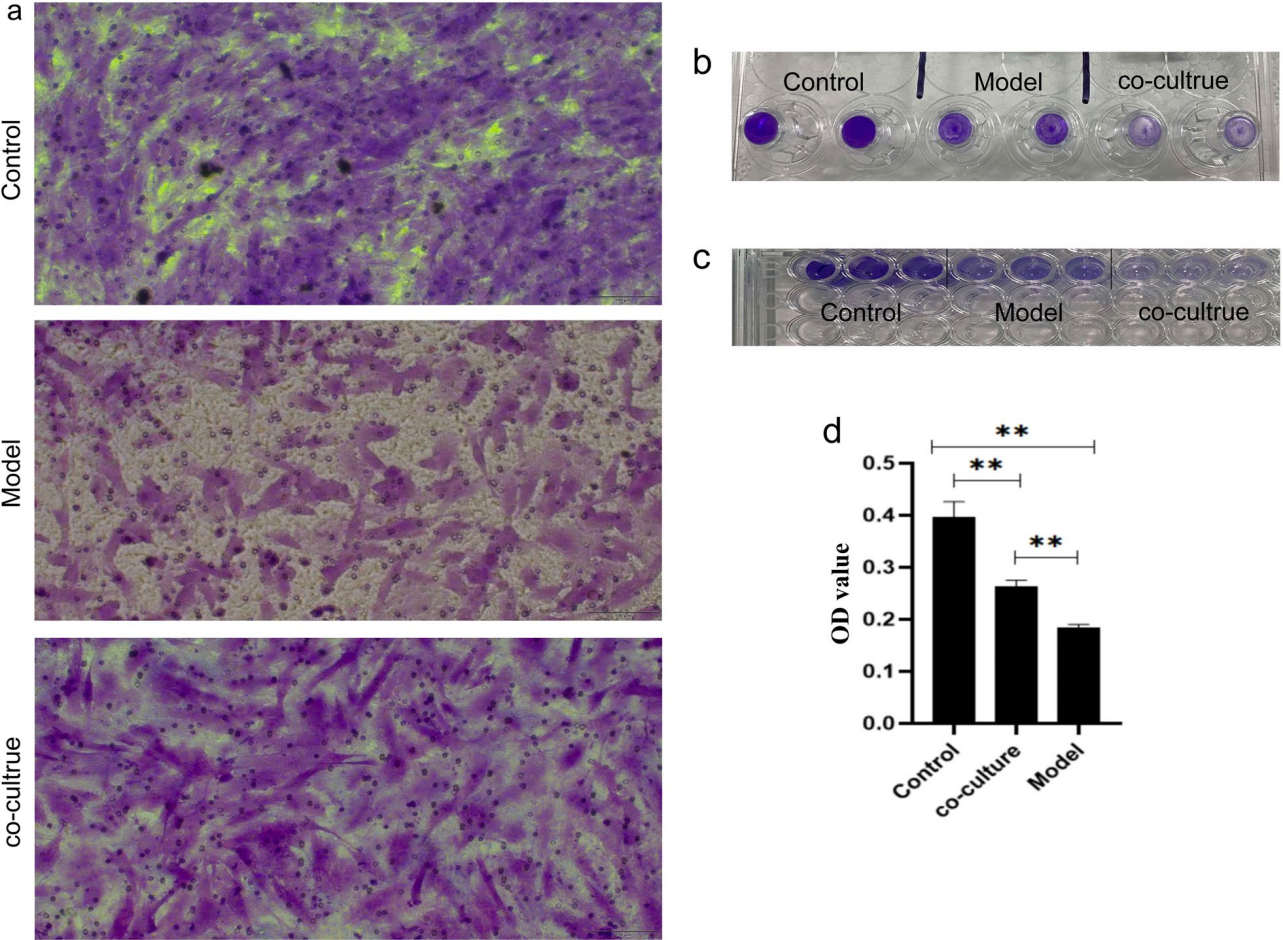
hGC cell migration experiment. **a** 200X field of vision; **b** stained polycarbonate film; **c** was transferred to a 96-well plate after staining and elution; and **d** OD statistics

The results of the cell migration experiment showed that the absorbance value measured using the enzyme label was higher in the coculture group than in the aging group (*p* < 0.01) (Fig. 15).

#### Verification of changes in upregulated gene expression levels of the MAPK signaling pathway in single-cell sequencing results

After coculture of aging hGCs and HA-MSCs, quantitative PCR detection of hGC MAPK pathway genes showed that the expression of these genes was upregulated compared with that in aging hGCs after coculture, which further explained the mechanism of HA-MSC treatment of ovarian aging and made hGCs younger.

**Fig. 16:**
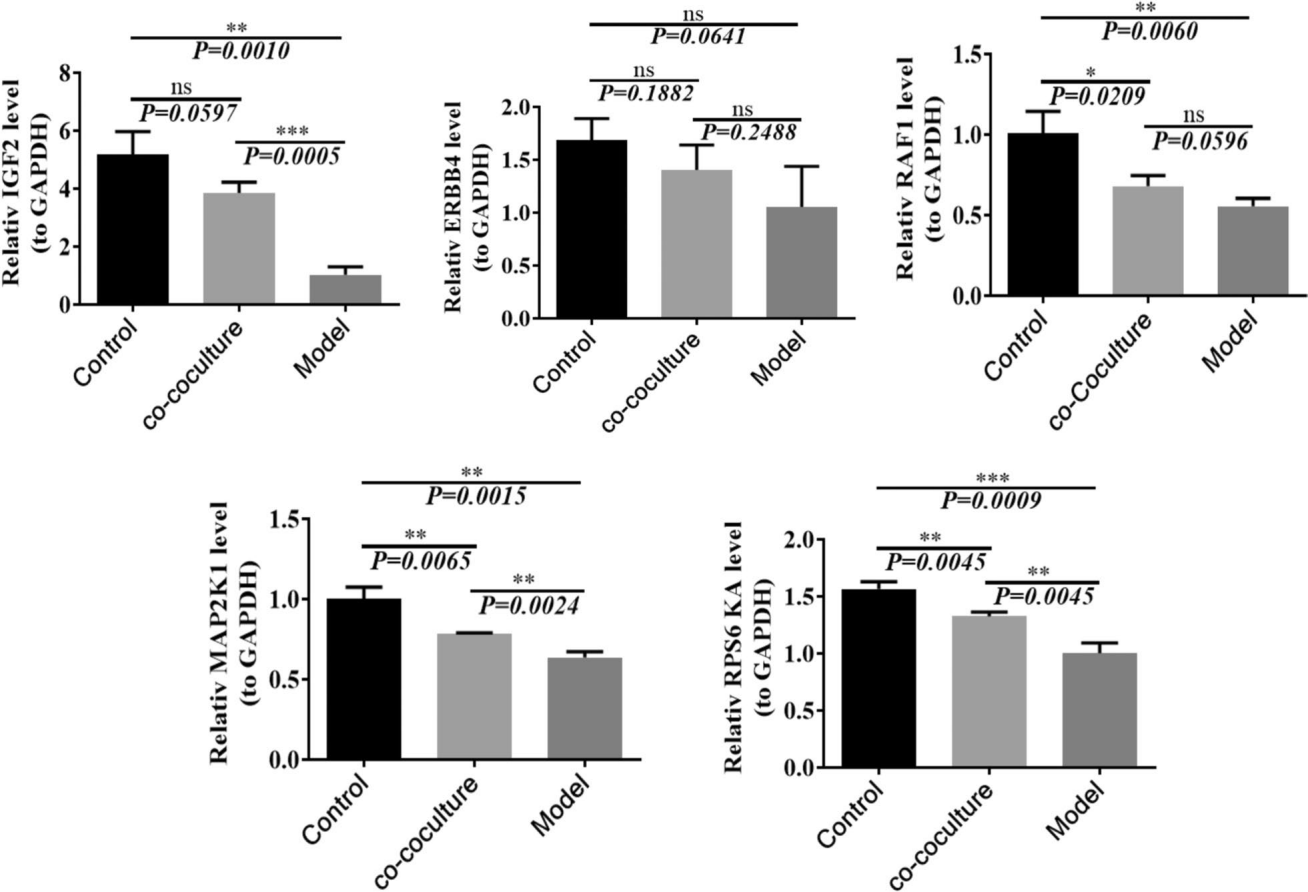
Changes in the expression levels of related genes in the MAPK signaling pathway. **a** Changes in the expression level of IGF2; **b** changes in ERBB4 expression level; **c** changes in the expression level of RAF1; **d** changes in MAP2K1 expression level; and **e** changes in the expression level of RPS6KA

The qPCR results showed that the expression levels of the IGF2, RAF1, MAP2K1, and RPS6kA genes in the MAPK signaling pathway were higher in the coculture group than in the aging group (*p* < 0.01) (Fig. 16).

These results indicate that after noncontact HA-MSC coculture with senescent hGCs, HA-MSCs can reverse the cell structure, proliferation ability, senescence condition, expression levels of senescence-related genes, and expression levels of key genes regulating the senescence pathway in the direction of normal hGCs.

## Discussion

To date, there have been many studies on the treatment of ovarian aging by stem cells, but they mainly use small animals such as mice, rats, or rabbits as models, and few studies use nonhuman primates. Nonhuman primates, represented by macaques, are highly similar to humans in terms of the menstrual cycle and hormone spectrum, so they have long been used as an ideal model for studies of human endocrinology and reproduction [22, 23]. Macaques have a lifespan of approximately 30 years and lose fertility at approximately 20–22 years [24]. The previous studies by our group have successfully established a standard ovarian aging model of macaque monkeys and found that after transplantation of HA-MSCs, the number of primary and secondary follicles of aging macaques significantly increased, and the degree of fibrosis of aging tissues significantly improved. The serum anti-Mullerian hormone (AMH) level is higher than that in MSCs. These results have preliminarily demonstrated the advantages of using HA-MSCs in resistance to ovarian aging. Furthermore, we used eGFP and SPION double labeling methods. Magnetic resonance imaging (MRI) technology was used to study the distribution, colonization, and outcome of stem cells in the body. HA-MSCs colonized and survived in ovarian tissue, which explained that HA-MSCs could indeed nest in ovarian tissue and directly participate in the repair of ovarian tissue. It was also speculated that HA-MSCs may not differentiate into follicles. Instead, ovarian function and structure are restored by the differentiation of HA-MSCs into granulosa cells. To further explore the cellular and molecular mechanisms of HA-MSCs in the treatment of ovarian senescence and to clarify the regulatory mechanism of resistance to ovarian senescence, ovarian tissues were collected after euthanasia of rhesus monkeys, and single nuclear transcriptome sequencing was performed.

The samples submitted for examination in this study were six tissues in two groups: three in the aging model group and three in the HA-MSC-treated group. However, one sample in each of the two groups failed the quality inspection due to possible problems in the sampling process or transportation process. The final sequencing samples were four in two groups: two in the aging model group and two in the HA-MSC-treated group.

The purpose of using male macaques was to see if the older females would mate and regain fertility after the treatment, although the older females did not become pregnant during the study period. However, one of the older females had another menstrual period after menopause. Six elderly female macaques were divided into two groups of three: one for the aging model group and one for the HA-MSC-treated group.

The results of single-cell RNA-seq analysis of macaque monkey ovaries showed that different ovarian cell subsets with varying transcription characteristics were identified according to the differential expression of different cell-specific markers. The correlation between the two cell subsets was calculated to determine the corresponding clusters of each cell, and different ovarian cell types were identified. Finally, eight cell clusters were identified as eight types of ovarian cells. They are stromal cells, endothelial cells, granular cells, theca cells, T cells, epithelial cells, smooth muscle cells, and monocytes. Except that no oocytes were found, the results were consistent with the study of Wang et al., in which they compared aging and normal macaque ovaries [25]. However, no significant changes in cell subsets or numbers were observed in the aging model group or the HA-MSC-treated group. Therefore, the sampling site, degradation during transportation, or the hypothesis that differentiation into granulosa cells rather than oocytes may be the reason for this phenomenon.

We analyzed the biological function of each cell cluster by using the differences between the differentially expressed gene scRNA-seq groups, revealing the unique characteristics of these ovarian cells. The results showed that the proportion and expression of cells containing the age-related genes p53, p21, and p16 were lower in the HA-MSC-treated group. The proportion and expression levels of cells containing the anti-aging genes GF, Sirt6, and GH were higher in the HA-MSC-treated group, indicating that after HA-MSC treatment, ovarian aging could be inhibited by the upregulation of anti-aging genes and the downregulation of age-related genes. In addition, 3755 upregulated genes were mainly distributed in endothelial cells, granulosa cells, and monocytes. It is speculated that the therapeutic effect of HA-MSCs on senescent ovaries is mainly to positively regulate the functions of endothelial cells, granulosa cells, and monocytes, including participation in the construction and remodeling of the vascular system. This promotes oocyte nutrition and maturation and inhibits the occurrence and development of inflammation.

To more intuitively display the distribution characteristics of genes in different samples and cell subsets, we selected the top 5 genes with multiple differences in each cluster according to their change in expression. The results showed that among the genes with significant differences, after HA-MSC treatment, the genes that were highly expressed were mainly GPSM, PAPPA2, and FGF, and other genes related to growth factors, protein synthesis regulation, and growth and development. In the aging model group, the genes that were highly expressed were mainly FOSB, HS3ST2, and FSTL4, and other genes related to tumors, cancer, and other diseases. The results indicated that HA-MSC therapy mainly activated growth- and development-related genes and inhibited the expression of disease-related genes at the gene level.

By the significance of pathway enrichment, we identified the major biochemical metabolic pathways and signal transduction pathways in which differentially expressed genes are involved. The KEGG results mainly focused on the biological metabolic pathways of organismal systems, genetic information processing, and cellular processes. According to the statistics of the top 20 pathways with the smallest Q values, the ribosome and MAPK signaling pathways with the largest number of pathways accounted for the number of all differentially expressed genes and the smallest Q values. The ribosome and MAPK signaling pathways are the most important biochemical metabolic pathways and signaling pathways in which differentially expressed genes are involved after HA-MSC treatment. In addition, changes in the size and size subunits of ribosomes are all upregulated. The expression of GF, RTK, Raf1, MEK1, and RSK2 in the MAPK signaling pathway was significantly upregulated. These results indicated that HA-MSC treatment could prevent ovarian senescence by affecting the biological functions of ribosomes and regulating the five key genes in the MAPK pathway.

qPCR results showed that the expression levels of GF (IGF2), Raf1 (RAF1), MEK1 (MAP2K1), and RSK2 (RPS6 KA) in the MAPK signaling pathway were upregulated in aging hGCs after coculture with HA-MSCs, and the results were consistent with those of single-cell sequencing. These results indicate that the MAPK signaling pathway is one of the key signaling pathways for HA-MSCs.

In summary, we explored the mechanism of HA-MSC treatment of ovarian aging at the molecular level through single-nucleus transcriptome sequencing and carried out validation experiments in vitro to further corroborate the results of single-cell sequencing, providing a reference scheme for the cellular and molecular mechanism of HA-MSC products in the treatment of ovarian aging. This work is expected to provide evidence for a new option in the treatment of ovarian senescence with stem cells and provide a new technical method and scientific basis for the clinical treatment of ovarian senescence.

## Conclusions

Through 10X Genomics single nuclear transcriptome sequencing, we investigated the mechanism of HA-MSC treatment on ovarian aging at the cellular and molecular levels and confirmed that HA-MSC treatment can improve the tissue structure and secretion function of the ovary and jointly play a role in anti-ovarian aging through multiple cellular and molecular mechanisms.

Validation experiments were carried out in vitro to further support the results of single-cell sequencing. These results provide a reference scheme for the cellular and molecular mechanism of HA-MSC products in the treatment of ovarian aging, a new choice for stem cell treatment of ovarian aging, and a new technical method and scientific basis for the clinical treatment of ovarian aging.

## Data Availability

All data generated or analyzed during this study are included in this published article. The original data have been uploaded to the GSA database. The assigned accession of the submission is: CRA013961. Please access it from the following link: https://bigd.big.ac.cn/gsa/browse/CRA013961.

## Abbreviation

MSCs: Mesenchymal stem cells
HA-MSCs: Highly active MSCs
GO: Gene Ontology
hGCs: Human ovarian granulosa cells
AMH: Anti-Mullerian hormone
MRI: Magnetic resonance imaging
IGF2: GF
RAF1: Raf1
MAP2K1: MEK1
RPS6 KA: RSK2
